# *Ptpn11* Deletion in CD4^+^ Cells Does Not Affect T Cell Development and Functions but Causes Cartilage Tumors in a T Cell-Independent Manner

**DOI:** 10.3389/fimmu.2017.01326

**Published:** 2017-10-16

**Authors:** S. M. Shahjahan Miah, Chathuraka T. Jayasuriya, Alexander I. Salter, Emma C. Reilly, Céline Fugere, Wentian Yang, Qian Chen, Laurent Brossay

**Affiliations:** ^1^Department of Molecular Microbiology and Immunology, Graduate Program in Pathobiology, Division of Biology and Medicine, Brown University Alpert Medical School, Providence, RI, United States; ^2^Department of Orthopaedics, Rhode Island Hospital and Brown University Alpert Medical School, Providence, RI, United States

**Keywords:** Src homology region 2 domain-containing phosphatase-2, T cells, invariant natural killer T cells, cartilage tumor, chondrocytes

## Abstract

The ubiquitously expressed tyrosine phosphatase Src homology region 2 domain-containing phosphatase-2 (SHP-2, encoded by *Ptpn11*) is required for constitutive cellular processes including proliferation, differentiation, and the regulation of immune responses. During development and maturation, subsets of T cells express a variety of inhibitory receptors known to associate with phosphatases, which in turn, dephosphorylate key players of activating receptor signaling pathways. We hypothesized that SHP-2 deletion would have major effects on T cell development by altering the thresholds for activation, as well as positive and negative selection. Surprisingly, using mice conditionally deficient for SHP-2 in the T cell lineage, we show that the development of these lymphocytes is globally intact. In addition, our data demonstrate that SHP-2 absence does not compromise T cell effector functions, suggesting that SHP-2 is dispensable in these cells. Unexpectedly, in aging mice, *Ptpn11* gene deletion driven by CD4 Cre recombinase leads to cartilage tumors in wrist bones in a T cell-independent manner. These tumors resemble miniature cartilaginous growth plates and contain CD4-lineage positive chondrocyte-like cells. Altogether these results indicate that SHP-2 is a cartilage tumor suppressor during aging.

## Introduction

Src homology region 2 domain-containing phosphatase-2 (SHP-2) is ubiquitously expressed and is required for gastrulation, hematopoiesis, and embryonic development (1, 2). SHP-2’s widespread expression positions it to affect the outcome of many cellular processes and human diseases. In humans, gain-of-function mutations in SHP-2 result in Noonan syndrome, a severe disease marked by dysmorphic facial features, short stature, heart disease, skeletal malformation, webbed neck, and mental retardation (3, 4). SHP-2 has also been postulated to function as a tumor suppressor, whereby diminished protein expression increases the risk for hepatocellular carcinoma (5). Additionally, mutation in *Ptpn11* has been shown to cause metachondromatosis, a rare syndrome leading to cartilage tumors (6).

Because SHP-2 is required for gastrulation, knockout mice die during gestation and conditional knockout animals must be used to study its effects in specific cell subsets. SHP-2 has been shown to both positively and negatively regulate cellular responses, depending on its interactions with other proteins; however, most reports suggest that SHP-2 plays a positive role in hematopoiesis (3, 7–10). SHP-2 has also been shown to act through the Ras pathway to promote cell proliferation (3). Nevertheless, SHP-2 has inhibitory functions in other contexts. For example, SHP-2 can inhibit the mTOR pathway and cell-cycle entry of activated T cells (11). SHP-2 is also believed to negatively regulate multiple JAK/STAT pathways, although the mechanism through which it acts remains unclear (3, 12). SHP-2 contains two tandem SH2 domains and a protein tyrosine phosphatase domain. The N-terminal SH2 domain mediates the binding of SHP-2 to other signaling proteins. At the steady state, SHP-2 activity is repressed due to the intra-molecular association of the N-terminal SH2 domain with the tyrosine phosphatase domain (13). Importantly, SHP-2 associates with immunoreceptor tyrosine-based inhibitory motifs on natural killer cell receptors (NKRs), further suggesting an inhibitory role (14).

Several subsets of T cells express inhibitory receptors known to associate with SHP-2. For instance, activated classical effector CD8^+^ T lymphocytes express members of the Ly49 receptor family, KLRG1, and PD-1 in addition to an αβ T cell receptor (TCR) (15). Invariant natural killer T (iNKT) cells also express certain inhibitory receptors (CD94/NKG2A, and members of the Ly49 family) in addition to a semi-invariant αβ TCR, which recognizes glycolipid antigens presented by the non-classical MHC class I molecule CD1d (16). Given that SHP-2 is implicated in TCR signaling and is known to associate with inhibitory NKRs expressed on CD8^+^ T cells and iNKT cells, we hypothesized that SHP-2 deletion would have major effects on development by altering the thresholds for activation, as well as positive and negative selection.

Both conventional T cells and iNKT cells develop in the thymus from shared T cell precursors. During maturation, T cell precursors pass through four double-negative (DN) stages, marked by an absence of CD4 or CD8 surface expression. As these immature cells proliferate, they begin to express CD4 and CD8 and enter the double-positive (DP, CD4^+^CD8^+^) stage of conventional T cell development. iNKT cells branch off from the DP stage following TCR α chain rearrangement and positive selection by CD1d-expressing CD4^+^CD8^+^ cortical thymocytes (17). Because conventional T cells and iNKT cells derive from DP thymocytes, we crossed mice with *loxP*-flanked alleles of *Ptpn11* with CD4-Cre transgenic and Lck transgenic lines to delete SHP-2 in both subsets. Unexpectedly, we found that SHP-2 is dispensable for the development, differentiation, and functions of both effector CD8^+^ T cells and iNKT cells.

Surprisingly, in aging mice, *Ptpn11* gene deletion driven by CD4 Cre recombinase (but not LckCre) led to cartilage tumors presenting large chondrocyte-like cells and fibrochondrocyte-like cells. Importantly, SHP-2^fl/fl^-CD4-Cre mice on a RAG null background also developed cartilage tumors, ruling out contributions from T cells. In support of this conclusion, we found that CD4-Cre was not restricted to T cells and was active in cartilage as well as several non-T cell subsets. Therefore, SHP-2 regulates cartilage homeostasis through a CD4-lineage positive subset.

## Materials and Methods

### Mice

Inbred C57BL/6 mice and B6.Cg-Tg(CD4-cre)1Cwi mice were purchased from Taconic Farms (Hudson, NY, USA). Inbred C57BL/6 mice, B6.SJL-Ptprca Pepcb/BoyJ, B6.Cg-Tg(Lck-cre)3779Nik/J, B6.129S7-Rag1^Tm1Mom^/J and B6.129P2-*Ptpn6^tm1Rsky^*/J (SHP-1^fl/fl^) were purchased from The Jackson Laboratory. *Ptpn11*^fl/fl^ (SHP-2^fl/fl^) mice with *LoxP* recombination sites flanking *Ptpn11* were previously described (18) and bred with CD4-Cre mice and Lck-Cre mice. SHP-2^fl/fl^-CD4-Cre mice were crossed to R26R-EFYP mice to produce SHP-2^fl/fl^-CD4-Cre-ROSA^EYFP^ mice. Jα18^−/−^ mice were bred, crossed to B6 (>12 generations). SHP-1^fl/fl^ mice were bred with CD4-Cre recombinase mice. All mice were bred in pathogen-free breeding facilities at Brown University. All experiments were conducted in accordance with institutional guidelines for animal care.

### Murine Lymphocyte Isolation

Mice were sacrificed by isofluorane treatment. Cardiac puncture was performed prior to the harvesting of the organs. Liver was perfused before harvesting. Spleens were dissociated and lymphocytes were enriched using Lympholyte Cell Separation Media (Cederlane). Livers were dissociated using a gentleMACS Dissociator (Miltenyi Biotec) and lymphocytes were enriched using a 40–70% discontinuous Percoll gradient (GE Healthcare) as previously described (19). Thymi were dissociated and thymocytes were washed prior to analysis.

### Surface Staining, Antibodies, and Flow Cytometry

Cells were stained with fluorochrome-conjugated monoclonal antibodies and 2.4G2 blocking antibody and incubated in the dark for 30 min on ice. For CD1d tetramer staining, cells were incubated in the dark for 15 min at room temperature followed by 15 min on ice. Flow cytometric analysis was performed on a FACSAria (BD Biosciences) or a MACSQuant (Miltenyi Biotec). Data were analyzed using FlowJo (Tree Star).

TCRβ-FITC, HSA-FITC, CD4-FITC, TCRβ-PE, Ly49C/I/F/H-PE, TCRβ-PerCPCy5.5, Ly49G2-PerCPeFluor710, CD62L-PE-Cy6, NK1.1-PE-Cy7, KLRG1-APC, CD25-APC, CD19-PE, CD44-APCeFluor780, CD8α-eFluor450, and CD3-eFluor450 were purchased from ebioscience. Ly49G2-FITC, Ly49C/I-FITC, Ly49A/D-PE, and CD4-PerCP were purchased from BD Pharmingen. BV421-CD127, BV510-TCRβ, BV570-CD45, BV605-CD3, BV605-CCR6, and BV785-NK1.1 were purchased from Biolegend. PE- and APC-conjugated α-GalCer loaded CD1d tetramer was prepared in our laboratory. CD1d, M45 and M57 tetramers were kindly provided by the National Institute of Allergy and Infectious Disease MHC Tetramer Core Facility at Emory University (Atlanta, GA, USA).

### Western Blot

Splenocytes from SHP-2 heterozygous (fl/+) and knockout (fl/fl) animals were sorted using a FACSAria to greater than 95% efficiency. TCRβ^+^ and TCRβ^−^ populations were collected and lysed in Lysis buffer. Cellular lysates were added to SDS/sample buffer, boiled, and run on a Ready Gel 4–20% Tris-HCl (Bio-Rad, Hercules, CA, USA) with 1× SDS-PAGE running buffer. The gel was blotted against a nitrocellulose membrane (Bio-Rad) using 1× transfer buffer with 10% methanol. After blocking in a 5% milk solution for 1 h at room temperature, rabbit α-mouse SHP-2 (Abcam) and β-actin (Ambion) antibodies were incubated with the membrane overnight at 4°C. HRP-conjugated-donkey α-rabbit IgG secondary antibody (Jackson Immunoresearch, Westgrove, PA, USA) was incubated with the membrane for 1 h at room temperature. Membranes were developed with Super signal Chemiluminescent Substrate (Pierce) and photographed using a ChemiDoc XRS + System (Bio-Rad).

### Microcomputed Tomography (μ-CT) and X-Ray Analysis

X-ray images of the entire skeleton were obtained immediately after euthanasia using a Faxitron X-ray system (Wheeling). After fixation in 4% paraformaldehyde, μ-CT images of skeletal tissues were scanned with a desktop microcomputer graphic imaging system (μ-CT40, Scanco Medical AG). For μ-CT, 3D image acquisitions took 36 min with a beam parameter of 70 kVp.

### Histology

Mice were euthanized at the indicated ages, and femurs, tibiae, and paws were removed and fixed in 4% paraformaldehyde overnight at 4°C. Postnatal skeletal tissues were decalcified in 0.5 M EDTA before embedding. Tissue sections (5 µm) were stained with hematoxylin and eosin. Some sections were separately stained with Safranin O and Fast Green.

### Murine Cytomegalovirus (MCMV) Infection

American Type Culture Collection Strain #VR1399 and RVG-102 clone of MCMV recombinant for GFP under the promoter of the immediate early gene-1 (i.e., 1) (a gift of Dr. Hamilton, Duke University) were prepared from salivary glands as previously described (20, 21). On day 0, mice were infected intraperitoneally with 5 × 10^4^ plaque-forming units (pfu) of MCMV. Mice were sacrificed for analysis at 7 days and 8–12 weeks postinfection.

### *In Vitro* iNKT Cell Stimulation and Cytokine Analysis

A method described by Stankovic et al was used (22). Briefly, CD1d tetramer-positive cells were sorted using a FACSAria to greater than 95% efficiency. A 96-well plate was coated with 0.5 μg/well purified α-mouse CD3ε and CD28 antibodies (eBioscience) and iNKT cells were plated at a concentration of 10,000 cells per well. Supernatant was collected 24 h poststimulation. Cytokine production was quantified by Cytometric Bead Array (BD Biosciences). Samples were run on a FACSAria and data were analyzed using FCAP Array Software (BD Biosciences).

### Proliferation Analysis

Recipient mice were non-lethally irradiated with 7.5 Gy and placed on oral Sulfamethoxazole and Trimethoprim (Hi-tech Pharmacal). On day 3 postirradiation, donor mice were sacrificed, thymi were harvested, and thymocytes were extracted and counted. CD8^+^ T cells were depleted using anti-CD8 magnetic beads *via* an AutoMACS. Remaining cells were stained for 10 min at 37°C in the dark with Cell Proliferation Dye eFluor450 (eBioscience) in PBS at a labeling concentration of 10 µM. 8–10 million cells were injected into each irradiated recipient. Six days postinjection, mice were sacrificed and the spleen and liver were harvested. Lymphocytes were extracted, stained, run on a MACSquant, and analyzed using FlowJo.

### Statistical Analysis

Data sets were analyzed for statistical significance using unpaired, two-tailed, Student’s *t*-tests. Values of *p* < 0.05 were considered significant. Graphs and statistics were generated and calculated using Prism (GraphPad).

## Results

### SHP-2 Is Dispensable for T Cell Development

**Figure 1:**
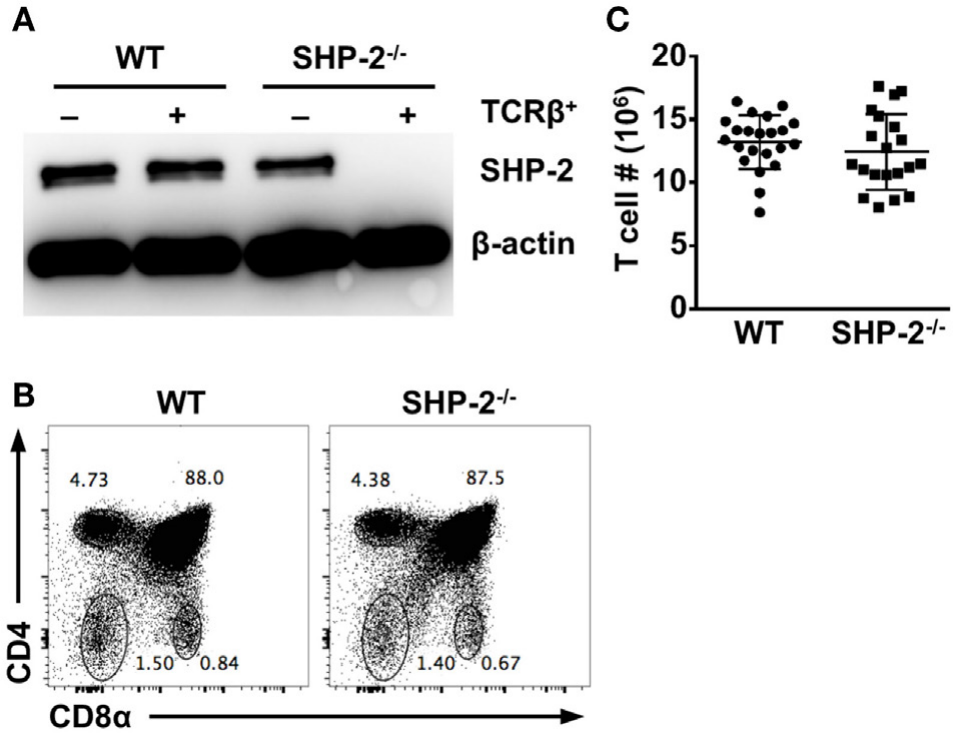
Src homology region 2 domain-containing phosphatase-2 (SHP-2) deficiency does not affect T cell development. **(A)** SHP-2 expression in splenocytes was evaluated by Western blot. YFP^+^CD3^+^TCRβ^+^ and YFP^−^CD3^−^TCRβ^−^ splenocytes were sorted from wild-type (WT) and SHP-2^−/−^ mice. Cellular lysates were blotted with α-SHP-2 and anti-β-actin antibodies. **(B)** Representative FACS plots show thymic T cell development in WT and SHP-2^−/−^ mice. **(C)** Number of T cells in the spleen of SHP-2 deficient and littermate controls.

SHP-2 floxed/floxed (fl/fl) mice (18) were crossed to mice carrying a transgene for Cre recombinase driven by the *cd4* enhancer/promoter/silencer (23, 24). As a result, the *Ptpn11* gene encoding SHP-2 is deleted at the DN4/DP stages of T cell development when CD4 is first expressed. For simplicity, we will refer to SHP-2^fl/fl^-CD4-Cre as SHP-2^−/−^ and their littermate controls, SHP-2^fl/+^-CD4-Cre, as wild type (WT). To verify that SHP-2 was efficiently deleted in T cells, splenic T cells and non-T cell populations from WT and SHP-2^−/−^ animals were sorted for protein analysis by Western blot using α-mouse SHP-2 primary antibody. Expression of SHP-2 in T cells was strongly reduced to undetectable levels SHP-2^−/−^ mice (Figure 1A).

We then analyzed thymic T cell development in our conditional knockout mice. We found that WT and SHP-2^−/−^ mice had comparable frequencies of DP (CD4^+^CD8^+^), single-positive (CD4^+^ or CD8^+^), and DN (CD4^−^CD8^−^) thymic lymphocytes (Figure 1B). Furthermore, no obvious T cell defects were observed in peripheral compartments. The number of splenic T cells (Figure 1C), splenic CD8^+^ T cells (Figures S1A,B in Supplementary Material) and CD4^+^ T cells (data not shown) were comparable between SHP-2^−/−^ mice and WT controls. However, not all T cells express inhibitory receptors, so the absence of SHP-2 could potentially affect the development of small subsets of T cells known to express these receptors. These include iNKT cells, Ly49^+^CD8^+^ T cells with a memory phenotype (CD44^hi^CD122^+^Ly6C^+^) (25), and exhausted T cells during chronic infection (26). To this end, we first examined CD8^+^ T cells with a memory phenotype by staining for CD44 and CD62L. No significant difference in the frequency of effector memory (CD44^hi^CD62L^lo^) CD8^+^ T cells between WT and SHP-2^−/−^ mice was observed (Figure S1C in Supplementary Material). SHP-2-deficiency also did not affect the frequency of KLRG1^+^ effector CD8^+^ T cells in all organs we tested (Figure S1D in Supplementary Material). Ly49 antibody cocktail staining revealed that Ly49 (Ly49C/I/F/H) expression did not substantially differ in the spleen, blood, or liver (Figure S1E in Supplementary Material). When analyzed separately, only in the case of the Ly49G2 receptor did we find a modest decrease of hepatic Ly49G2^+^ CD8^+^ T cells in SHP-2^−/−^ mice (Figure S1F in Supplementary Material).

As previously mentioned, iNKT cells also express a variety of inhibitory receptors such as Ly49 (27). While the role of inhibitory receptors on iNKT cells has not been defined, it has been suggested that they control iNKT cell autoreactivity and tolerance (28, 29). Due to the association of these receptors with SH2 domain-containing phosphatases, we hypothesized that the absence of SHP-2 might affect iNKT cell development. Because iNKT cells are positively selected by CD1d-expressing CD4^+^CD8^+^ cortical thymocytes (30), SHP-2 is absent in both iNKT cell precursors and DP cells mediating positive selection in our conditional knockout mice. However, CD1d tetramer staining showed no significant difference in the absolute number of iNKT cells (Figures S2A,B in Supplementary Material). Additionally, no defects were observed in iNKT cell frequency among each stage of thymic maturation, where the majority of cells progressed to mature Stage III iNKT cells (Figure S2C in Supplementary Material). The TCR Vβ chain repertoire and Ly49 expression profile of iNKT cells from WT and SHP-2^−/−^ mice were also comparable (data not shown). Collectively, these results indicate that SHP-2 is dispensable during iNKT cell development and maturation.

**Figure 2:**
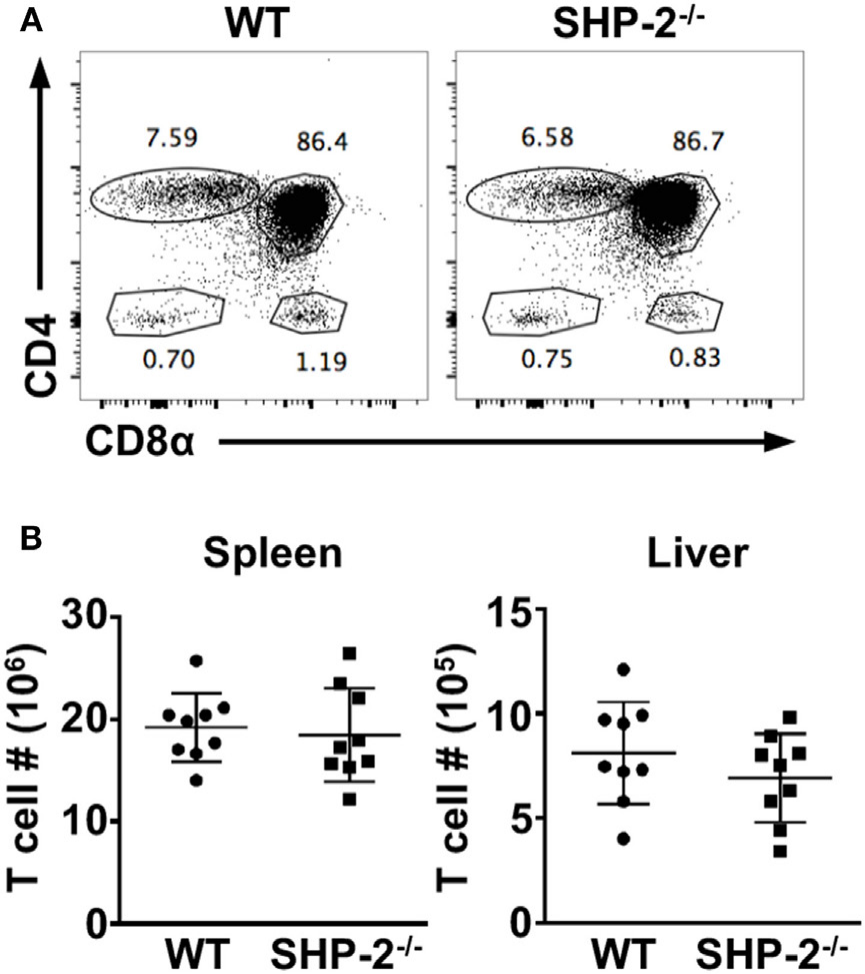
T cell development is mostly normal in SHP-2^−/−^LCK-Cre mice. **(A)** Representative FACS plots show thymic T cell development in wild-type (WT) and SHP-2^−/−^ mice. **(B)** Number of T cells in the spleen and liver of SHP-2^−/−^ mice and littermate controls. Data are pooled from three independent experiments, *n* = 9. Error bars indicate SD.

It has been previously reported that conditional deletion of SHP-2 by placing Cre recombinase under the control of *Lck* promoter results in defective conventional T cell development (8). As we did not find a T cell defect when SHP-2 was deleted by CD4-Cre, we also crossed SHP-2^fl/fl^ mice to *Lck*-Cre transgenic mice, another T cell-specific Cre recombinase mouse line. Similarly to the SHP-2^fl/fl^-CD4-Cre mice, no developmental defect was observed in T cells in the thymus (Figure 2A) or in the periphery (Figure 2B). Altogether the data using both SHP-2^fl/fl^-CD4-Cre mice and SHP-2^fl/fl^-Lck-Cre mice demonstrate that SHP-2 is globally dispensable for T cell development.

### SHP-2 Is Not Required for iNKT Cell Effector Functions and Effector CD8^+^ T Cell Differentiation

We next sought to determine whether deletion of SHP-2 affected iNKT cell and T cells functions. Because activated iNKT cells are known to rapidly secrete large amounts of cytokines upon activation, we quantified cytokine production following *in vitro* TCR cross-linking as previously described (22). Normalization of three independent experiments revealed that iNKT cell cytokine production was unaffected by SHP-2 absence (Figure S2D in Supplementary Material). More specifically, IL-2, IL-4, IFN-γ, and TNF-α production by SHP-2-deficient iNKT cells did not differ significantly from WT controls. To assess TCR-mediated cytokine production in an *in vivo* setting, we injected WT and SHP-2^−/−^ mice intraperitoneally with 2 µg of α-GalCer. α-GalCer is the prototypical ligand for iNKT cells and leads to vigorous cytokine production and proliferation (20). Because iNKT cell TCR cell surface expression is reduced after activation, we analyzed iNKT cells at early time points. At 2 h poststimulation, intracellular cytokine staining showed similar frequencies of IL-4^−^IFN-γ^−^, IL-4^+^, IFN-γ^+^, and IL-4^+^IFN-γ^+^ iNKT cells between WT and SHP-2^−/−^ mice (Figure S2E in Supplementary Material). To evaluate whether the absence of SHP-2 causes an iNKT cell proliferative defect, we intravenously injected CD8-depleted SHP-2^−/−^ (CD45.2^+^) and congenic WT (CD45.1^+^) thymocytes into sublethally irradiated Jα18^−/−^ mice. Proliferation cycles were quantified by Cell Proliferation Dye eFluor450. In these experiments, SHP-2^−/−^ and congenic WT iNKT cells proliferated to an equal extent in the leukopenic environment (Figure S2F in Supplementary Material). These results suggest that SHP-2 does not affect iNKT cell ability to proliferate.

**Figure 3:**
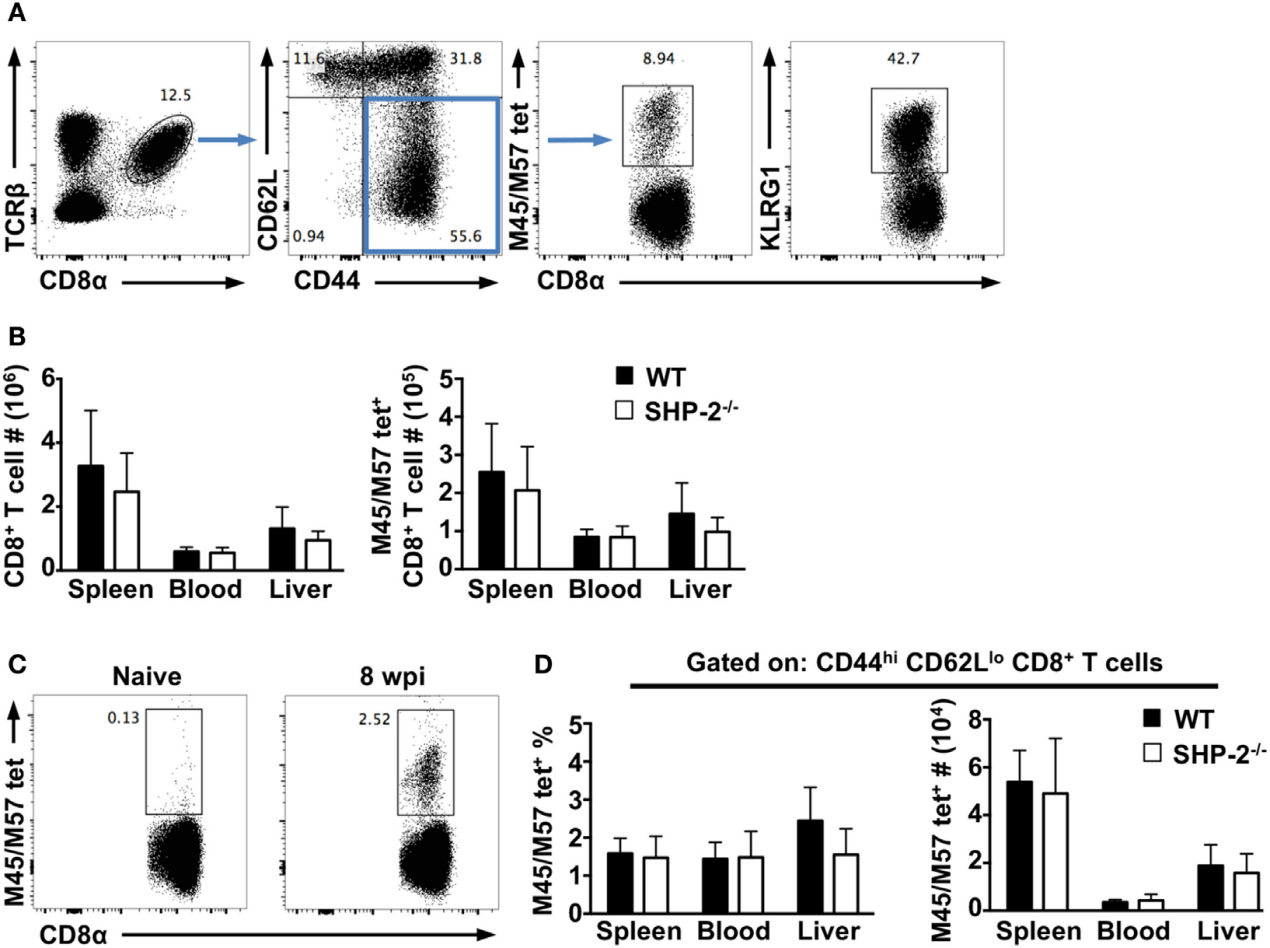
Src homology region 2 domain-containing phosphatase-2 (SHP-2) does not affect early immune responses and memory CD8^+^ T cell differentiation during murine cytomegalovirus (MCMV) infection. **(A)** Representative FACS plots show staining of CD8^+^ T cells and m45/57tet^+^ CD8^+^ T cells at day 7 postinfection. **(B)** The absolute numbers of CD8^+^ T cells and m45/57tet^+^ CD8^+^ T cells at day 7 postinfection. *n* = 10 wild-type (WT) and 8 SHP-2^−/−^ mice over two independent experiments. Graphs show mean and SD. **(C)** Representative FACS plots show staining for antigen-specific CD8^+^ T cells in naïve and infected WT mice. **(D)** The absolute number and frequency of m45/57tet^+^ CD44^hi^CD62L^lo^ CD8^+^ T cells from the spleen, blood, and liver of mice at 10 weeks post infection. *n* = 5 WT and 5 SHP-2^−/−^ mice. Graphs show mean and SD.

We next asked whether SHP-2 affected the formation of effector CD8^+^ T cells during infection. Using the MCMV infection model, we examined CD8^+^ T responses at early and late time points (7 days and 8–12 weeks postinfection, respectively). We found the frequencies of CD8^+^ T cells, CD44^hi^CD62L^lo^ effector memory CD8^+^ T cells, and KLRG1^+^CD8^+^ T cells to be nearly identical between WT and SHP-2^−/−^ mice at both time points (Figure 3). Several MCMV epitopes have been described in C57BL/6 mice including M38, M139, M45, and M57 (21). CD8^+^ T cells specific to M38 and M139 are classified as inflationary cells (31), while CD8^+^ T cells specific for M45 and M57 epitopes have been shown to differentiate into a classical central memory phenotype (31, 32). We simultaneously examined CD8^+^ T cells specific for M45 and M57 epitopes, which expand during acute infection and have been associated with long-term protection against MCMV (21). On day 7 postinfection, no significant difference in the number of effector Ag-specific CD8^+^ T cells was observed (Figure 3B). Furthermore, at 10 weeks postinfection, we found that SHP-2 deficiency did not affect the formation of Ag-specific memory CD8^+^ T cells (Figure 3C,D). Overall, the data suggest that SHP-2 is dispensable for Ag-specific CD8^+^ T cell formation. Interestingly, preliminary analysis of SHP-1^fl/fl^-CD4-Cre conditional mice indicates that there is no gross defect in T cell and iNKT cell development (Figure S3 in Supplementary Material), suggesting a possible redundancy between SHP-1 and SHP-2 in T cell development. Generation of mice conditionally deficient for both SHP-1 and SHP-2 in the T cell lineage will clarify this issue.

### Adult SHP-2^fl/fl^-CD4-Cre Mice Develop Cartilage Tumors

**Figure 4:**
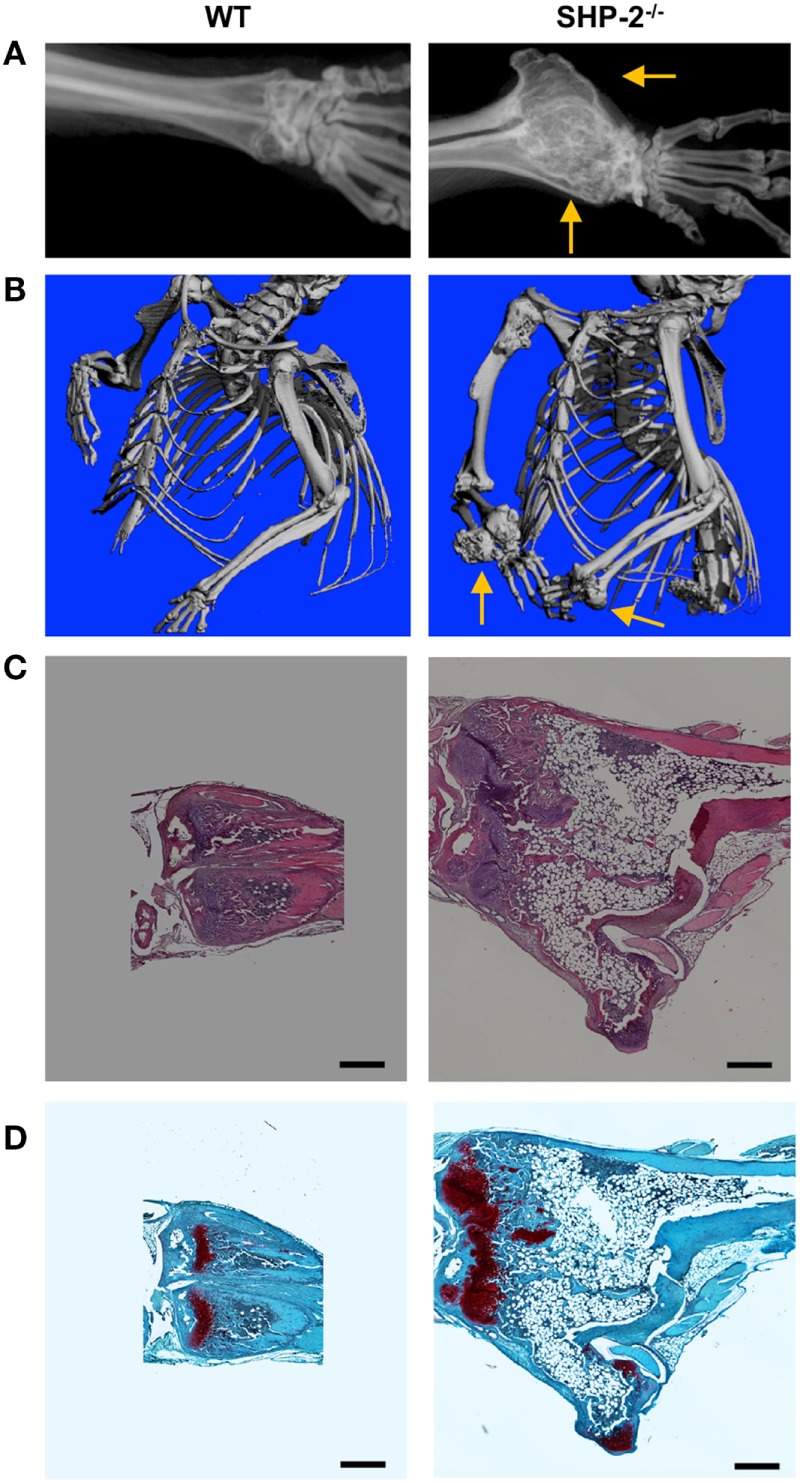
SHP-2^−/−^ mice develop cartilage tumors. **(A)** Representative Faxitron X-ray of euthanized mice, 9 months of age. Arrows highlight the location of the wrist. **(B)** Representative microcomputed tomography (μ-CT) analysis shows the wrist tumor in SHP-2^−/−^ mice at 9 months of age. **(C)** Representative hematoxylin and eosin staining of distal radius of 6-month-old mice. **(D)** Representative Safranin-O/Fast Green staining of distal radius of 6-month-old mice reveals abnormal cartilaginous regions within the wrist tumor of SHP-2^−/−^ mice. Each analysis was performed on at least three mice per genotype. Scale bar represents 500 µm.

Since SHP-2 is a regulator of lymphocyte activation, we hypothesized that aging SHP-2-deficient mice might be predisposed to autoimmune disease, as reported for other T cell tyrosine protein phosphatases (33–35). Although we did not observe any signs of autoimmunity (data not shown), we surprisingly found that 6 month-old SHP-2^fl/fl^-CD4-Cre mice exhibited markedly decreased mobility, while littermate controls did not. Faxitron X-ray radiographs and μ-CT of SHP-2^−/−^ mice revealed enlarged bony masses at the wrists (Figures 4A,B) and knees (data not shown). Moreover, X-rays showed kinky tails (Figure S4A in Supplementary Material) and spinal abnormalities (Figure S4B in Supplementary Material) in most SHP-2^−/−^ animals, likely due to overgrowth of the bones in the vertebral column (data not shown). We analyzed a larger group of mice (>30 WT and 30 SHP-2^−/−^) and found this phenotype was gender independent and exhibited 100% penetrance in SHP-2-deficient animals at 5 months of age. Faxitron radiographs indicated that the enlarged bony masses at the wrists were not detectable before 4 months of age (data not shown). H&E staining of the distal radius showed significant differences in bone morphology and revealed that SHP-2^−/−^ mice appear to have cartilage tumors resembling miniature growth plates (Figure 4C). The wrist tumor consists of large areas of cartilage tissue (1–3), indicated by positive Safranin O staining (Figure 4D). Based on their morphology and staining, the cells in these cartilaginous regions appear to be a combination of large chondrocyte-like cells and fibrochondrocyte-like cells, which is not observed in the heterozygote littermate controls. Normal bone growth and development rely on coordination between chondrocyte mass regulation and cartilage structure establishment. Overall, the phenotype observed is reminiscent of metachondromatosis. To this end, both heterozygous *Ptpn11* frame shift mutations and deletions in chondroid cells have been shown to cause metachondromatosis (36), suggesting that *Ptpn11* is a tumor suppressor gene in cartilage.

### Cartilage Tumor Development Is T Cell Independent

The previous experiments utilized SHP-2^−/−^ mice in which floxed *Ptpn11* was deleted by Cre recombinase under the control of the *cd4* promoter/enhancer/silencer. However, other mammalian cell lineages, including lymphoid tissue-inducer (LTi) cells, have also been reported to express CD4 during development and the observed phenotype could potentially be explained by CD4 expression outside of the T cell compartment (37, 38). In support of this hypothesis, SHP-2^fl/fl^-Lck-Cre mice did not develop cartilage tumors suggesting that the phenotype observed was T cell independent (data not shown). We then bred SHP-2^fl/fl^-CD4-Cre mice on the RAG^−/−^ background. In the resultant SHP-2^fl/fl^-CD4-Cre-RAG^−/−^ mice, cartilage tumors developed (Figure S5 in Supplementary Material). The cartilage tumors in these mice can be detected at approximately the same age as the Shp-2^fl/fl^-CD4-Cre mice. Because SHP-2^fl/fl^-CD4-Cre-RAG^−/−^ mice do not have T cells, we concluded that the phenotype observed is T cell independent. Altogether, these data demonstrate that *Ptpn11* gene deletion in a non-T cell subset leads to cartilage tumors.

**Figure 5:**
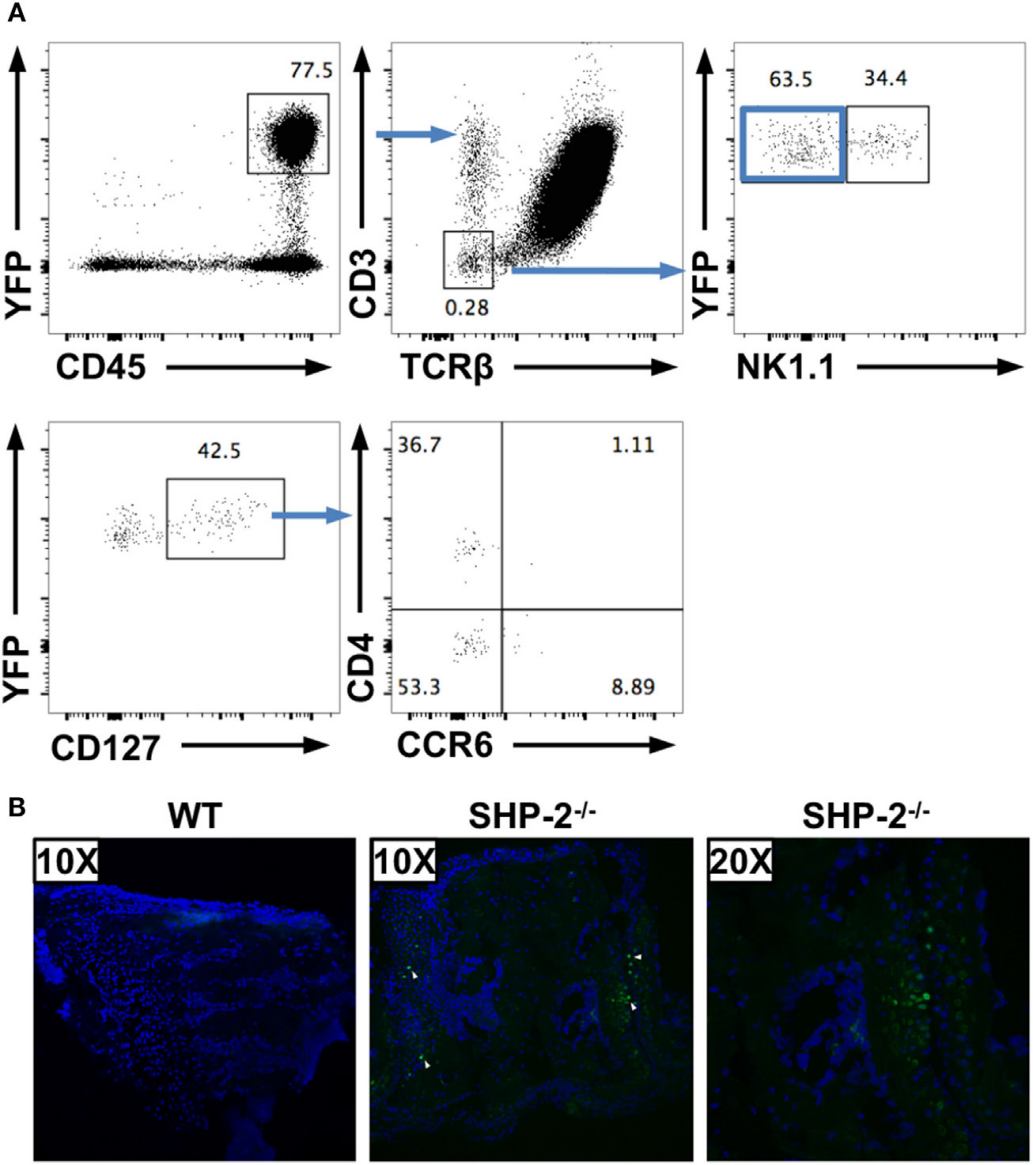
Several non-T cell subsets have an history of CD4 expression. **(A)** CD19^−^YFP^+^ splenocytes were gated for non-T cells (CD3^−^TCRβ^−^) and separated in NK1.1^−^ and NK1.1^+^ cells. CD127^+^ cells from NK1.1^−^ cells were subdivided based on CD4 and CCR6 expression. **(B)** 30 µm serial sections of frozen wrist tissues were prepared from SHP-2^±^CD4-Cre-RosaYFP and SHP-2^−/−^CD4-CreRosaYFP mice and imaged on a Nikon C1SI confocal microscope. Nuclei indicated by DAPI staining (blue) and YFP detected in GFP channel (green, white arrows).

To mark cells that have deleted the *Ptpn11* gene, we crossed the SHP-2^fl/fl^-CD4-Cre mice to RosaYFP reporter mice (39). In the resultant SHP-2^fl/fl^-CD4-Cre-RosaYFP mice, Cre activity simultaneously deletes the *ptpn11* gene locus and removes a loxP-flanked stop cassette to drive constitutive expression of a YFP gene. This fate mapping strategy allowed us to identify SHP-2-deficient cells even if they no longer express CD4. Using these mice, we found that a low number of CD45^+^CD19^−^CD3^−^TCRβ^−^ cells are YFP positive in spleens (Figure 5A), lymph nodes (data not shown), and liver (data not shown) of SHP-2^fl/fl^-CD4-Cre-RosaYFP mice. The proportion of these cells are low and appear to derive from different lineages. For instance, in the spleen a subset of the CD45^+^CD3^−^ CD19^+^ cells (not shown) and a subset of the CD45^+^CD3^−^ NK1.1^+^ cells express YFP (Figure 5A). In addition, within the YFP^+^ population, a CD3^−^CD19^−^NKp46^−^NK1.1^−^CD127^+^ subset can be identified, which can be subdivided into CD4^+^ and CD4^−^ cells (Figure 5A). These cells are reminiscent of LTi-like cells (37, 38); however, only a few of these cells express CCR6 (Figure 5A).

Importantly, we have been able to detect few YFP-positive cells in frozen wrist sections of aged Shp-2^fl/fl^-CD4-Cre-RosaYFP mice by confocal microscopy (Figure 5B). No YFP-positive cells were found in frozen sections from the control heterozygote mice (Figure 5B), indicating that localization of the cells of interest are concomitant to the wrist tumors. These data confirm that several non-T cell subsets express CD4 or had expressed CD4 during development. In the spleen, we have shown that SHP-2 is efficiently deleted from YFP-positive cells (Figure 1), indicating that one of these subsets could be responsible for the phenotype observed.

## Discussion

Src homology region 2 domain-containing phosphatase-2 is known to affect the quality of immune responses through its association with NK cell inhibitory receptors where the phosphatase is thought to dephosphorylate key players of cellular activation (9, 40, 41). However, inhibitory receptor-independent inhibition of cytolytic activity and IFN-γ secretion has also been reported in NK cells (42). Because iNKT cells and subsets of conventional T cells express inhibitory receptors, we hypothesized that SHP-2 deletion on T cells would have significant effects. Here, we report that conditional deletion of SHP-2 under the CD4 and Lck-distal promoters do not markedly affect conventional T cells and iNKT cells. This phenotype is relatively surprising given the well-documented necessity for SHP-2 during gastrulation, hematopoiesis, and embryonic development (3), as well as its reported association with the TCR immunoreceptor tyrosine-based activation motifs (43, 44). In both these conditional KO mice, thymic and peripheral T cell development, as well as effector CD8^+^ T cell differentiation and function, are comparable. iNKT cell development and function are also unaffected by a lack of SHP-2. Our results appear to contradict a previous report that used a different variant of SHP-2 conditional KO mice, where SHP-2 was deleted under the *Lck* promoter (8). In contrast to the authors’ findings, we did not detect any block in thymic T cell development. We also did not find reduced cellularity in the spleen and thymus or a decrease in TCR driven proliferation and cytokine production, as previously reported (8). Although we cannot explain the differences with the previous published study, another laboratory has found that T cell development is not affected by SHP-2 absence (Greta Guarda, personal communication).

The level of tyrosine phosphorylation and immune cell activation is determined by the opposing actions of cytoplasmic protein tyrosine kinases, phosphotyrosine phosphatases, and lipid phosphatases. Tyrosine phosphorylation is a reversible process, whereby phosphatases direct dephosphorylation of the tyrosyl residue. The SH2 domain-containing phosphotyrosine phosphatases SHP-2, and its related cellular tyrosine phosphatase SHP-1, have been shown to play essential roles in immune cell signaling (9, 45). Because our SHP-2^−/−^ mice do not display an apparent T cell phenotype, it is possible that SHP-2 and SHP-1 play redundant functions in regulating immune signaling. SHP-1 is well expressed in hematopoietic cells and has been shown to limit the threshold of T cell activation and other immunological processes (3, 46, 47). We initially wanted to cross SHP-1 conditional knockout mice to our SHP-2^−/−^. Unfortunately, due to the fact that the *Ptpn6* gene is located on chromosome 6, 4.2 megabases from the NK locus, there is no NK1.1 expression and the Ly49 repertoire is predominantly from 129 origin (data not shown) in the available mice (48), precluding the generation of double-deficient mice. Nevertheless, our preliminary analysis of SHP-1^fl/fl^-CD4-Cre conditional mice suggests that there is no gross defect in T cell and iNKT cell development, thus reinforcing a possible redundancy between SHP-1 and SHP-2 in T cell development.

The development of cartilage tumors in aged SHP-2^−/−^ mice was unexpected. Using a variety of genetic and cellular approaches, our findings indicate that these tumors result from loss of SHP-2 in a cell subset(s) expressing CD4 during their development. Although the precise identification of these cells is beyond the scope of this manuscript, our data using RAG^−/−^ and Lck-Cre transgenic mice rule out T cells as responsible for the phenotype. Using reporter mice for CD4 expression, we identified several non-T cell subsets that express YFP, a surrogate marker for prior CD4-Cre activity. Interestingly, low number of YFP^+^ cells are found in a variety or organs, including wrist sections. As mentioned above, LTi-like cells can express CD4 (37) and *CD4* RNA expression has been reported in chondrocytes (49). Therefore, CD4^+^ LTi-like cells and CD4^+^ chondrocytes are among the potential candidates responsible for the observed skeleton abnormalities.

A phenotype reminiscent of the tumors observed in the current study has been recently described in humans (6, 50). Using linkage analysis and parallel sequencing of bar-coded DNAs from several families, mutations in *Ptpn11* were identified as a cause of metachondromatosis, a rare tumor syndrome leading to cartilage tumors (6). Notably, although patients with metachondromatosis have one mutant copy and one normal copy of *Ptpn11* in all cells, the normal copy is lost in cartilage cells that form tumors (6), reinforcing the critical tumor suppressor role of SHP-2 in cartilages. In mice, it has been found that SHP-2 regulates the proliferation of Ctsk chondroid progenitors, which are responsible for cartilage growth (36). In another study, deletion of SHP-2 in mesenchymal stem cells, which are progenitors of both osteoblasts and chondrocytes, was shown to cause multiple skeleton abnormalities (51). However, in these two studies, the phenotype is more severe and occurs within 8 weeks of birth, while our SHP-2^−/−^ mice do not develop tumors before 4 months of age, suggesting that SHP-2 deletion occurs at a later stage of chondrocyte differentiation. In mice, using a mosaic postnatal inactivation of *Ptpn11*, it has been shown that SHP-2-deficient cells affect SHP-2^+^ chondrocytes, *via* a paracrine mechanism. This leads to a delay in terminal differentiation of growth plate chondrocytes (52). Additionally, the authors demonstrated that SHP-2-deficient fibroblast precursors can inappropriately differentiate into chondrocyte-like cells (52). We have been able to detect chondrocyte-like cells (Figure 4) and YFP^+^ cells (Figure 5) in the cartilage tumors. We therefore hypothesize that the SHP-2-deficient cell subset may either inappropriately differentiate into chondrocyte-like cells or cause neighboring SHP-2-sufficient cells to contribute to exostoses and enchondromas *via* paracrine signals. For instance, Bowen and colleagues showed that mosaic inactivation of *Ptpn11* in growth plates affect *Ptpn11* deficient and WT chondrocytes (52). Therefore, it is possible that SHP-2-deficient cells directly or indirectly activate mesenchymal stem cells to undergo chondrogenesis. Interestingly, a similar phenotype was recently reported when *Sos1/2* or *Erk1/2* were deleted from CD4^+^ cells (49, 53). Sos proteins are Ras-guanine exchange factors, which can be recruited downstream of SHP-2, and promote the activation of ERK. It is therefore likely that the SHP-2/SOS/ERK pathway is critically involved in the regulation of a subset of CD4^+^ cells, leading to cartilage tumors.

## Ethics Statement

This study was carried out in strict accordance with the recommendations in the Guide for the Care and Use of Laboratory Animals as defined by the National Institutes of Health (PHS Assurance #A3284-01). Animal protocols were reviewed and approved by the Institutional Animal Care and Use Committee (IACUC) of Brown University. All animals were housed in a centralized and AAALAC-accredited research animal facility that is fully staffed with trained husbandry, technical, and veterinary personnel.

## Author Contributions

SM, CJ, ER, and CF conceived, performed, and analyzed the experiments. AS conceived, performed, analyzed the experiments, and wrote the article. WY provided reagents. QC conceived and analyzed the experiments. LB conceived, analyzed the experiments, and wrote the article. The authors have no conflict of interest to declare.

## Conflict of Interest Statement

The authors declare that the research was conducted in the absence of any commercial or financial relationships that could be construed as a potential conflict of interest.
