# Supplementary material for: Ptpn11 Deletion in CD4+ Cells Does Not Affect T Cell Development and Functions but Causes Cartilage Tumors in a T Cell-Independent Manner
Source: Front Immunol. 2017 Oct 16;8:1326. doi: 10.3389/fimmu.2017.01326 (PMC5650614; doi:10.3389/fimmu.2017.01326)
Supplement: Supplementary file 1 [file presentation_1.pdf]

Supplementary Figure 1

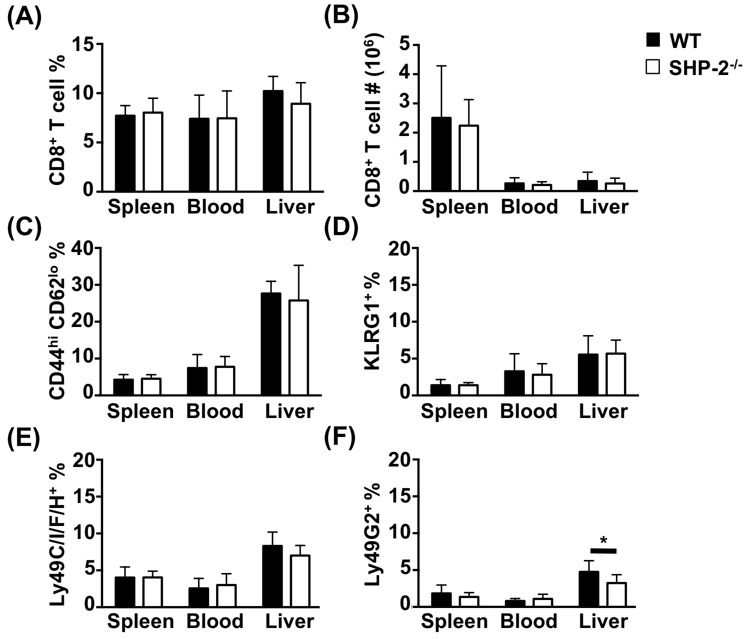

Supplementary Figure 1. SHP-2 deficiency does not affect T cell development. Absolute frequencies (A) and numbers (B) of $CD8^+$ T cells were determined by flow cytometry and cell counting. Frequencies of $CD44^{hi}CD62L^{lo}$ (C) $KLRG1^+$ (D), $Ly49C/I/F/H^+$ (E), and $Ly49G2^+$ (F) $CD8^+$ T cells from the spleen, blood, and liver of WT and SHP-$2^{-/-}$ mice were determined by flow cytometry. T cells were defined as $TCR\beta^+$ lymphocytes. n = 11 WT and 10 SHP-$2^{-/-}$ mice over three independent experiments. Graphs show mean and standard deviation. * = $p <$ 0.05.
