# Supplementary material for: Ptpn11 Deletion in CD4+ Cells Does Not Affect T Cell Development and Functions but Causes Cartilage Tumors in a T Cell-Independent Manner
Source: Front Immunol. 2017 Oct 16;8:1326. doi: 10.3389/fimmu.2017.01326 (PMC5650614; doi:10.3389/fimmu.2017.01326)
Supplement: Supplementary file 2 [file presentation_2.pdf]

Supplementary Figure 2

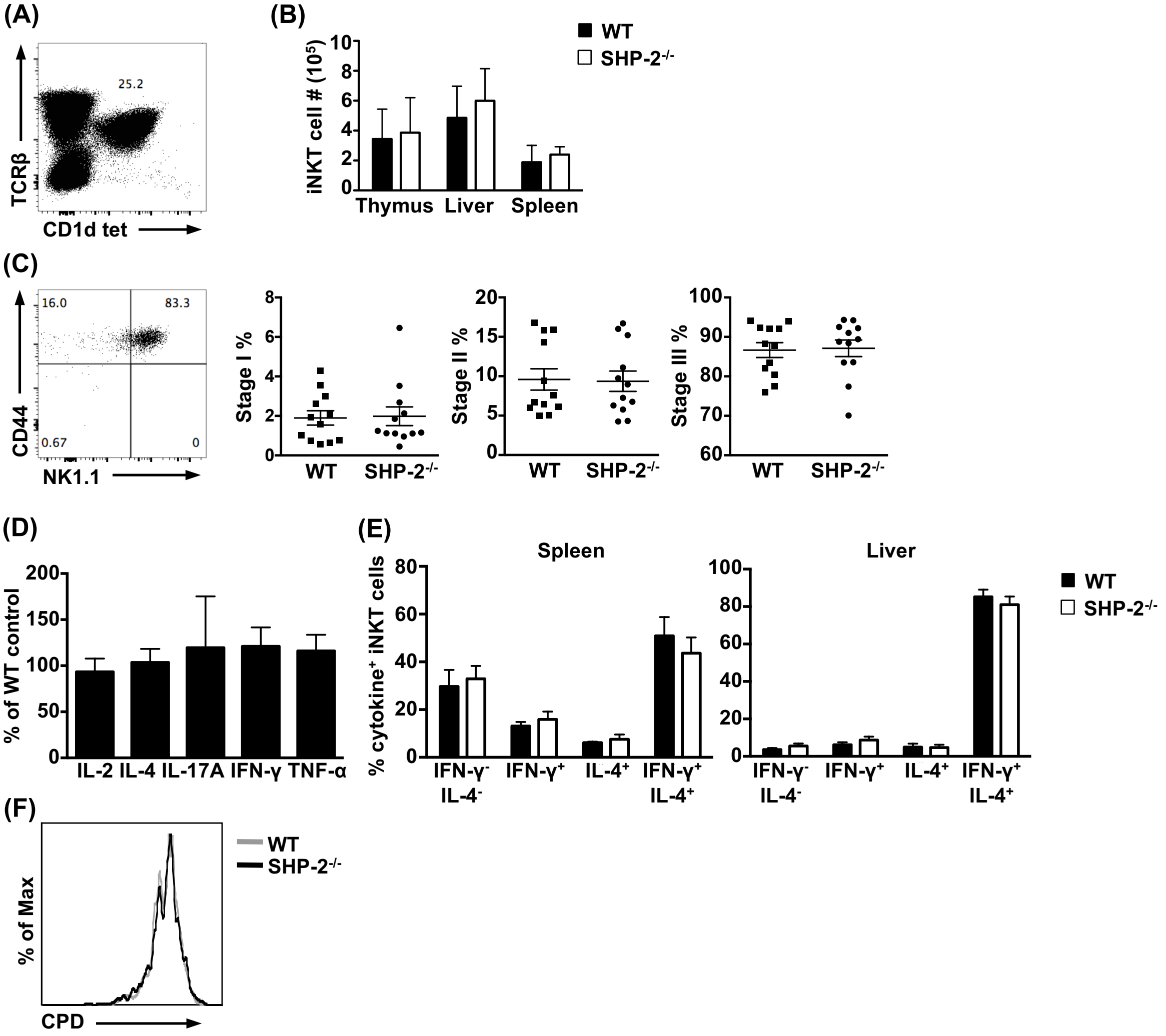

Supplementary Figure 2. SHP-2 deficiency does not affect iNKT cell development an functions. (A) Representative FACS plot shows hepatic iNKT cells in a WT mouse. (B) Absolute numbers of iNKT cells in WT and SHP-$2^{-/-}$ mice were determined by flow cytometry and cell counting. iNKT cells were defined as $HSA^{lo}CD1tet^{+}$ lymphocytes in the thymus and $TCR\beta^{+}CD1tet^{+}$ lymphocytes in the liver and spleen. n = 12 WT and 12 SHP-$2^{-/-}$ mice over four independent experiments. Graphs show mean. (C) Representative FACS plot shows thymic iNKT cell maturation in a WT mouse. Thymic iNKT cell maturation can be divided into three stages. Stage I (CD44loNK1.1-), Stage II (CD44hiNK1.1-), and Stage III (CD44hiNK1.1+) of development is shown in WT and SHP-$2^{-/-}$ mice. n = 12 WT and 12 SHP-$2^{-/-}$ mice over four independent experiments. Graphs show mean. (D) *In vitro* cytokine production was measured by CBA Flex Set after TCR cross-linking with α-CD3ε and α-CD28 antibodies for 24 hours. Graph shows mean and standard deviation of three independent experiments normalized to controls. (E) *In vivo* cytokine production was measured by intracellular cytokine staining at 2 hours post-injection with 2μg/mouse α-GalCer. n = 3 WT and 3 SHP-$2^{-/-}$ mice. Graphs show mean and standard deviation. (F) The proliferative capacity of iNKT cells was determined by injecting cells into a leukopenic environment. Representative FACS plot shows proliferation of SHP-$2^{-/-}$ ($CD45.2^{+}$, black) and WT ($CD45.1^{+}$, grey) iNKT cells ($CD1tet^{+}TCR\beta^{+}$) in the liver.
