# Supplementary material for: Ptpn11 Deletion in CD4+ Cells Does Not Affect T Cell Development and Functions but Causes Cartilage Tumors in a T Cell-Independent Manner
Source: Front Immunol. 2017 Oct 16;8:1326. doi: 10.3389/fimmu.2017.01326 (PMC5650614; doi:10.3389/fimmu.2017.01326)
Supplement: Supplementary file 3 [file presentation_3.pdf]

Supplementary Figure 3

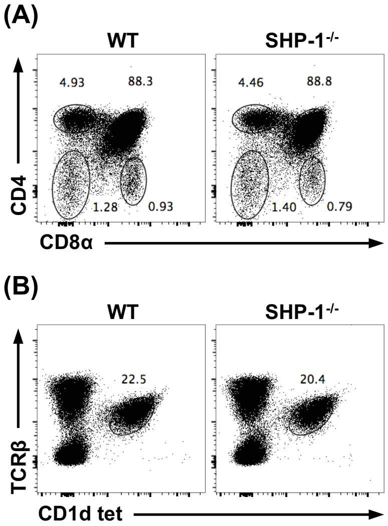

Supplementary Figure 3. SHP-1 is dispensable for T and iNKT cell development. (A) Representative FACS plots show thymic T cell development in SHP-1$^{fl/+}$-CD4Cre (WT) and SHP-1$^{fl/fl}$-CD4Cre (SHP-1$^{-/-}$) mice. (B) Representative FACS plots show hepatic iNKT cells in WT and SHP-1$^{-/-}$ mice. Data are representative of at least two independent experiments, n = 9 Het and n=8 KO mice.
