# Supplementary material for: Ptpn11 Deletion in CD4+ Cells Does Not Affect T Cell Development and Functions but Causes Cartilage Tumors in a T Cell-Independent Manner
Source: Front Immunol. 2017 Oct 16;8:1326. doi: 10.3389/fimmu.2017.01326 (PMC5650614; doi:10.3389/fimmu.2017.01326)
Supplement: Supplementary file 4 [file presentation_4.pdf]

Supplementary Figure 4

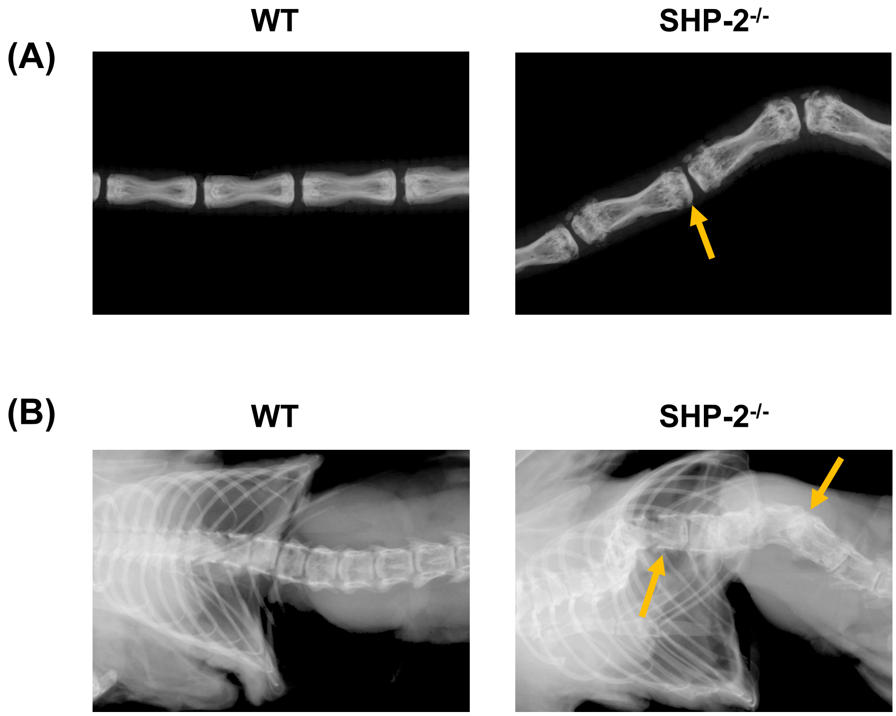

Supplementary Figure 4. Adult SHP-2$^{-/-}$CD4-Cre mice develop skeleton abnormalities. Representative Faxitron X-ray of euthanized mice. A) The arrow highlights the location of the kinky tails. B) Arrows highlight the spine abnormality. Each analysis was performed on at least 3 mice per genotype.
