# Supplementary material for: Ptpn11 Deletion in CD4+ Cells Does Not Affect T Cell Development and Functions but Causes Cartilage Tumors in a T Cell-Independent Manner
Source: Front Immunol. 2017 Oct 16;8:1326. doi: 10.3389/fimmu.2017.01326 (PMC5650614; doi:10.3389/fimmu.2017.01326)
Supplement: Supplementary file 5 [file presentation_5.pdf]

Supplementary Figure 5

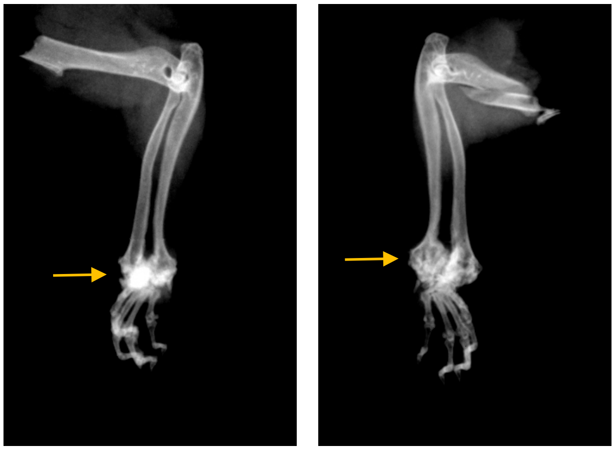

Supplementary Figure 5. Adult SHP-2$^{-/-}$CD4-Cre RAG1$^{-/-}$ mice develop cartilage tumors. Representative Faxitron X-ray of euthanized mice. Arrows highlight the location of the cartilage tumors on wrists. Analysis was performed on 3 SHP-2$^{-/-}$RAG$^{-/-}$ CD4-Cre mice and controls.
